# Unipro UGENE NGS pipelines and components for variant calling, RNA-seq and ChIP-seq data analyses

**DOI:** 10.7717/peerj.644

**Published:** 2014-11-04

**Authors:** Olga Golosova, Ross Henderson, Yuriy Vaskin, Andrei Gabrielian, German Grekhov, Vijayaraj Nagarajan, Andrew J. Oler, Mariam Quiñones, Darrell Hurt, Mikhail Fursov, Yentram Huyen

**Affiliations:** 1 Unipro Center for Information Technologies, Novosibirsk, Russia; 2 Bioinformatics and Computational Biosciences Branch, Office of Cyber Infrastructure and Computational Biology, National Institute of Allergy and Infectious Diseases, NIH, Bethesda, MD, USA

**Keywords:** Bioinformatics, Next-generation sequencing, Data analysis, ChIP-seq, Variant calling, RNA-seq

## Abstract

The advent of Next Generation Sequencing (NGS) technologies has opened new possibilities for researchers. However, the more biology becomes a data-intensive field, the more biologists have to learn how to process and analyze NGS data with complex computational tools. Even with the availability of common pipeline specifications, it is often a time-consuming and cumbersome task for a bench scientist to install and configure the pipeline tools. We believe that a unified, desktop and biologist-friendly front end to NGS data analysis tools will substantially improve productivity in this field. Here we present NGS pipelines “Variant Calling with SAMtools”, “Tuxedo Pipeline for RNA-seq Data Analysis” and “Cistrome Pipeline for ChIP-seq Data Analysis” integrated into the Unipro UGENE desktop toolkit. We describe the available UGENE infrastructure that helps researchers run these pipelines on different datasets, store and investigate the results and re-run the pipelines with the same parameters. These pipeline tools are included in the UGENE NGS package. Individual blocks of these pipelines are also available for expert users to create their own advanced workflows.

## Introduction

Running computational tools on cloud or high performance computing (HPC) environments is a popular way to cope with large amounts of next generation sequencing (NGS) data. For example, public instances of Galaxy (Goecks, Nekrutenko & Taylor, 2010) help biologists in managing tools and workflows by providing easy-to-use web interfaces. Galaxy also has broadly-used collections of NGS data analysis recipes. The Taverna suite (Wolstencroft et al., 2013) allows executing workflows that typically mix web services and local tools. Tight integration with myExperiment (Goble et al., 2010) gives Taverna access to a network of shared workflows, including NGS data processing ones. However, the use of publicly available instances is not always a viable solution due to the increasing size of input data. Transfer delays due to limits in bandwidth, especially in infrastructure poor environments, unnecessarily extend the time needed for analysis.

If a laboratory has its own infrastructure, the workflow systems can be installed locally; however, only a technical specialist can install a local instance because the systems have many dependencies and require additional configuration.

Desktop tools are useful for relatively small or medium scale in-house NGS analyses. In small labs or labs without bioinformaticians or servers, the biologist’s personal computer is typically powerful enough to handle different NGS-related analyses. Desktop tools’ use of local files helps avoid the data transfer issues, in contrast toweb-based tools that rely on remote file storage and Internet connectivity. To support productive research, desktop tools for NGS analysis should have an intuitive GUI and should be easy to install and configure by non-advanced computer users. Widely used command-line-based tools for variant, ChIP-seq and RNA-seq analyses are SAMtools (Li et al., 2009), MACS (Zhang et al., 2008) and TopHat (Trapnell, Pachter & Salzberg, 2009) correspondingly. Examples of GUI-based commercial desktop tools for generic NGS analyses are CLCBio (http://www.clcbio.com), Geneious (http://www.geneious.com) and Partek (http://www.partek.com/).

Unipro UGENE (Okonechnikov et al., 2012) is a desktop multiplatform open-source software package, integrating dozens of widely used bioinformatics tools and providing a rich graphical interface for various biological objects and associated tools. In particular, it integrates several tools for sequencing reads mapping: Bowtie (Langmead et al., 2009), BWA (Li & Durbin, 2009) and the original UGENE Genome Aligner tool. Assembled data can be visualized in the UGENE Assembly Browser. UGENE integrates popular bioinformatics tools and visualization capabilities in a single graphical interface with a common data model for several of these tools, unlike other open source tool kits that only provide the original tools without any value-addition.

Moreover, the UGENE toolkit allows one to automate common tasks by creating workflows with the integrated tools using the UGENE Workflow Designer. A brief comparison of the Workflow Designer with other workflow managing systems can be found in Supplemental Information 1 of the UGENE article (Okonechnikov et al., 2012).

## Implementation

The purpose of our work was to build a framework inside UGENE for NGS data analysis on a desktop computer that would cover three NGS data analysis areas: variant calling, RNA-seq data analysis, and ChIP-seq data analysis. To solve analysis tasks in each area, popular open-source tools were gathered into pipelines. The Workflow Designer was chosen as a basis to create the pipelines.

We also made sure that the NGS framework included all the necessary tools within it and that the pipelines are easy to use. In addition to running the pipelines, we also designed the framework with components that would allow the users to do manipulations such as: storing, managing, reproducing results of different runs of the tools, and obtaining information on input parameters or statistics.

### Providing availability

To make the pipelines available out of the box, all the required tools and data were added into the UGENE NGS package. This includes:

• *Variant calling pipeline:* tools from the SAMtools package (samtools and bcftools executables, the vcfutils script) and the perl interpreter.
• *RNA-seq pipeline:* tools from the Bowtie package (the aligner and the build index executables); tools from the Bowtie2 (Langmead & Salzberg, 2012) package (the aligner and the build index executables); TopHat (the main python script, etc.); tools from the Cufflinks (Trapnell et al., 2010) package (cufflinks, cuffcompare, cuffdiff, cuffmerge, and gffread) and the python interpreter. In addition to these tools, the pipeline uses the already mentioned SAMtools package.
• *ChIP-seq pipeline:* MACS (the main “macs14” and other python scripts) and other tools from Galaxy/Cistrome (Liu et al., 2011): CEAS (the main “ceas” and other python scripts), the Peak2Gene python script, the ‘Conservation Plot’ tool (the main “conservation_plot” and other python scripts), SeqPos (the main “MDSeqPos” python script and other files) and ConductGo (“go_analysis” python script and R utils); the python interpreter with numpy and django additional modules; the R environment (http://www.r-project.org/) with additional Bioconductor (http://www.bioconductor.org/) modules. The NGS package also includes internal data for the tools such as gene annotations, genome sequences and conservation scores.

In addition, some of the tools were ported to other operating systems (see the “Results” section for details).

### Developing user interface

There were already some capabilities in the Workflow Designer to simplify the creation and usage of workflows before the NGS framework project was started. For example, there was embedded context documentation for each workflow element and its parameters, ready embedded samples of different workflows and validation of a created workflow. Therefore, we used these capabilities for the NGS pipelines: we added context documentation for each component of each pipeline, added ready-to-use samples for the pipelines and improved validation of the pipelines.

Originally, a user of the Workflow Designer had to select a workflow element to set up its parameters. This procedure was simplified by providing a new wizards infrastructure, namely, a wizard was added to each sample NGS workflow allowing one to configure the workflow step-by-step.

### Simplifying the results analysis

Workflows in UGENE are stored in text files in the original UWL (UGENE Workflow Language) format. The format specifies the elements of a workflow, connections between the elements, parameters, and so on. It makes it possible to store a workflow with filled parameters and reuse it later.

Our idea was to go further in this direction and save the whole context of a workflow run, including the output files and logs of the run, and store it between different UGENE sessions. A new infrastructure to visualize the context and manage different results was developed. This was done by introducing “dashboards” which also show context statistics when a workflow is run.

## Results and Discussions

Below we describe the sample workflows that are used in UGENE by default for each type of covered NGS analysis. UGENE also allows one to create new workflows from the building blocks of the pipelines.

### Variant calling pipeline

**Figure 1:**
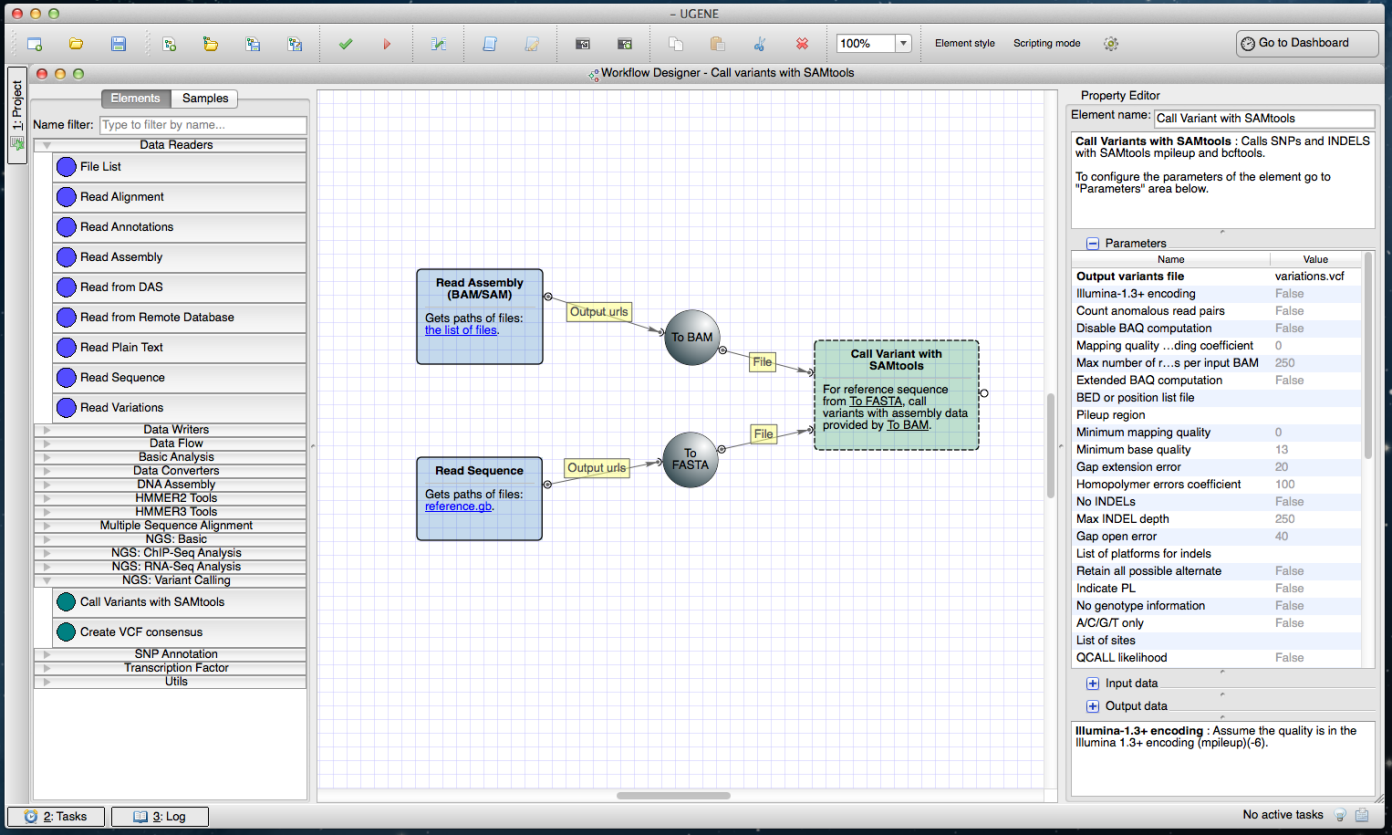
SAMtools workflow in a Workflow Designer window. The workflow itself can be seen at the center of the window on the Workflow Designer scene. The left side of the window shows available workflow elements (i.e., building blocks for a workflow), grouped by categories. The “NGS: Variant Calling” group, in particular, is opened. The right side of the window displays the description of the currently selected element “Call Variants”.

SAMtools utilities (mpileup, bcftools view, vcftutils) comprise the variant calling pipeline (https://ugene.unipro.ru/wiki/display/WDD/Call+Variants+with+SAMtools) (Fig. 1). In its original form SAMtools is a command-line Unix-based tool. The embedded version is multi-platform and has a GUI.

To run the pipeline a user inputs aligned reads (in SAM or BAM format (Li & Durbin, 2009)) and a reference sequence (in any sequence format supported by UGENE). The resulting variants may be produced in various formats for example, in VCF4 (http://www.1000genomes.org/node/101).

### RNA-seq pipeline

**Figure 2:**
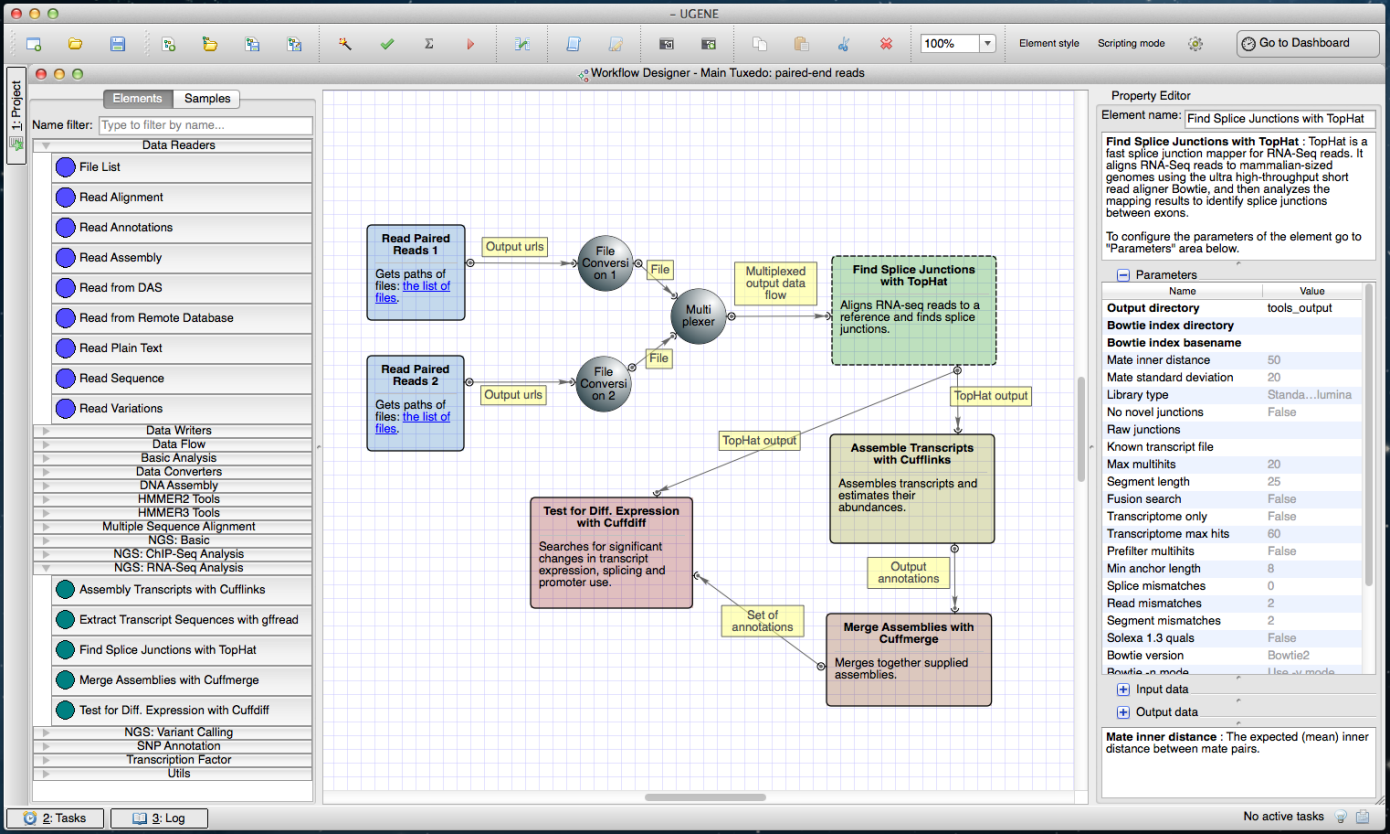
Tuxedo workflow in a Workflow Designer window. The workflow is shown at the center of the window on the Workflow Designer scene. This is a full version of the pipeline with paired-end reads used as input. The left side of the window shows building blocks available for building a new RNA-Seq analysis workflow. The right side of the window shows parameters of the selected “Find Splice Junctions with TopHat” element.

UGENE incorporates the Tuxedo protocol (Trapnell et al., 2012) for complex analysis of RNA-seq data: identification of new genes, splice junctions analysis and differential gene and transcript expression analysis. This protocol describes usage of TopHat and tools from the Cufflinks package(Cufflinks, Cuffdiff and Cuffmerge). The original protocol is based on a set of command-line tools; the UGENE version adds a GUI that connects them together. The pipeline (https://ugene.unipro.ru/wiki/display/WDD/RNA-seq+Analysis+with+Tuxedo+Tools) (Fig. 2) is available on Mac OS X and Linux operating systems.

Three versions of the sample Tuxedo workflow are available in UGENE: the “Full Tuxedo Pipeline” for analyzing and comparing two or more RNA-seq experiments, the “Single-sample Tuxedo Pipeline” for a single experiment analysis and the “No-new-transcripts Tuxedo Pipeline” for analysis without producing new transcripts.

All three workflows begin with TopHat, which takes a genome and RNA-seq short reads in BAM or SAM formats, aligns the reads to the genome and finds the splice junctions. In the full and single-sample pipelines, Cufflinks assembles the transcripts after TopHat. The full pipeline adds Cuffmerge to merge assemblies from different experiments. The last step of the full and no-new-transcript pipelines is the differential gene and transcript analysis performed by Cuffdiff.

### ChIP-seq pipeline

**Figure 3:**
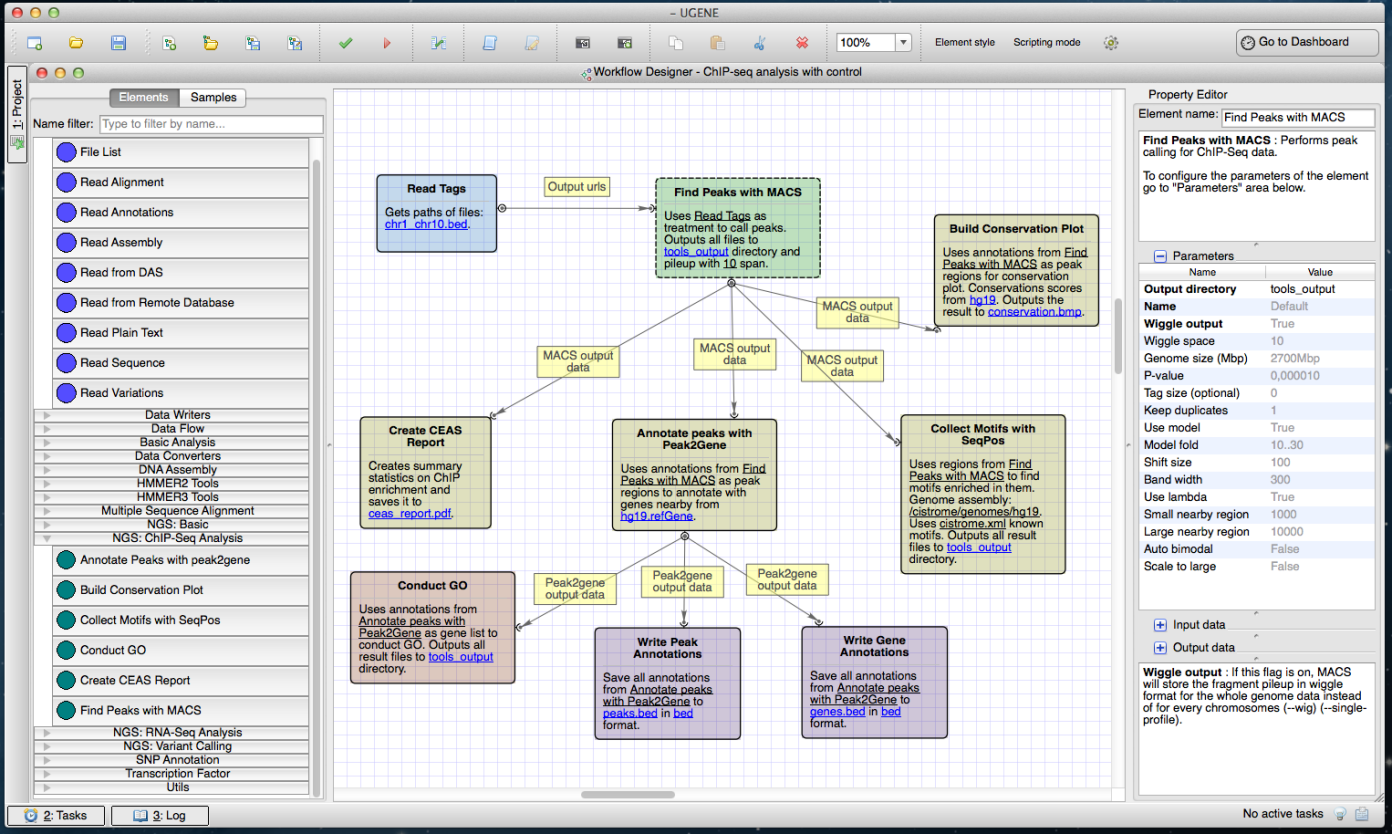
Cistrome workflow in a Workflow Designer window. The elements of the workflow can be seen at the center of the window on the Workflow Designer scene. Building blocks for a new ChIP-Seq analysis pipeline are shown on the left side of the window. A description of the currently selected element (“Find Peaks with MACS”) is shown on the right side of the window.

A general ChIP-seq analysis pipeline from the Cistrome platform was used as a guide to create the ChIP-seq pipeline in UGENE (https://ugene.unipro.ru/wiki/display/WDD/ChIP-seq+Analysis+with+Cistrome+Tools) (Fig. 3). The pipeline performs downstream analysis of ChIP-seq reads which involves finding target genes and motifs of TFBSs, and the annotation of functions of regulated genes. Original Cistrome is available on a Galaxy public instance. The UGENE NGS package incorporates tools from Cistrome and the package of internal data for the tools, such as gene annotations, genome sequences and conservation scores. The UGENE version of these tools is available on both Unix and Windows platforms.

The ChIP-seq analysis starts with peak-calling by MACS (Model-based Analysis of ChIP-seq) which takes ChIP-seq reads in BED (http://genome.ucsc.edu/FAQ/FAQformat.html#format1) format. MACS outputs peak regions and peak summits in BED format and, optionally, ChIP fragment pileup along the whole genome at every 10 bp (by default) in WIG (http://genome.ucsc.edu/FAQ/FAQformat.html#format6) format.

The pipeline passes the MACS output to other tools:

(1) CEAS generates a common report in PDF and a gene-centered annotation output in a tab-delimited text file with XLS extension.
(2) The SeqPos tool finds motifs that are enriched closed to the peak centers. SeqPos can use motifs from several databases: the human protein-DNA interaction database (hPDI) (Xie et al., 2009), JASPAR (Portales-Casamar et al., 2009), Protein Binding Microarray (PBM) (Zhu et al., 2009), TRANSFAC (Matys et al., 2006) and Yeast-1-hybrid (y1h) (http://www.clontech.com/). It can also find *de novo* motifs using the MDscan algorithm (Liu, Brutlag & Liu, 2002). The found motifs are output to a HTML page.
(3) The ‘Conservation Plot’ tool generates a figure showing the average conservation score profiles around the peak centers.
(4) Peak2Gene finds the nearest gene for each peak. It is followed by functional analyses of the found genes. The analyses are based on the association of Gene Ontology (GO) terms to genes. GOstats package written in R is used in this step.

### Wizards

**Figure 4:**
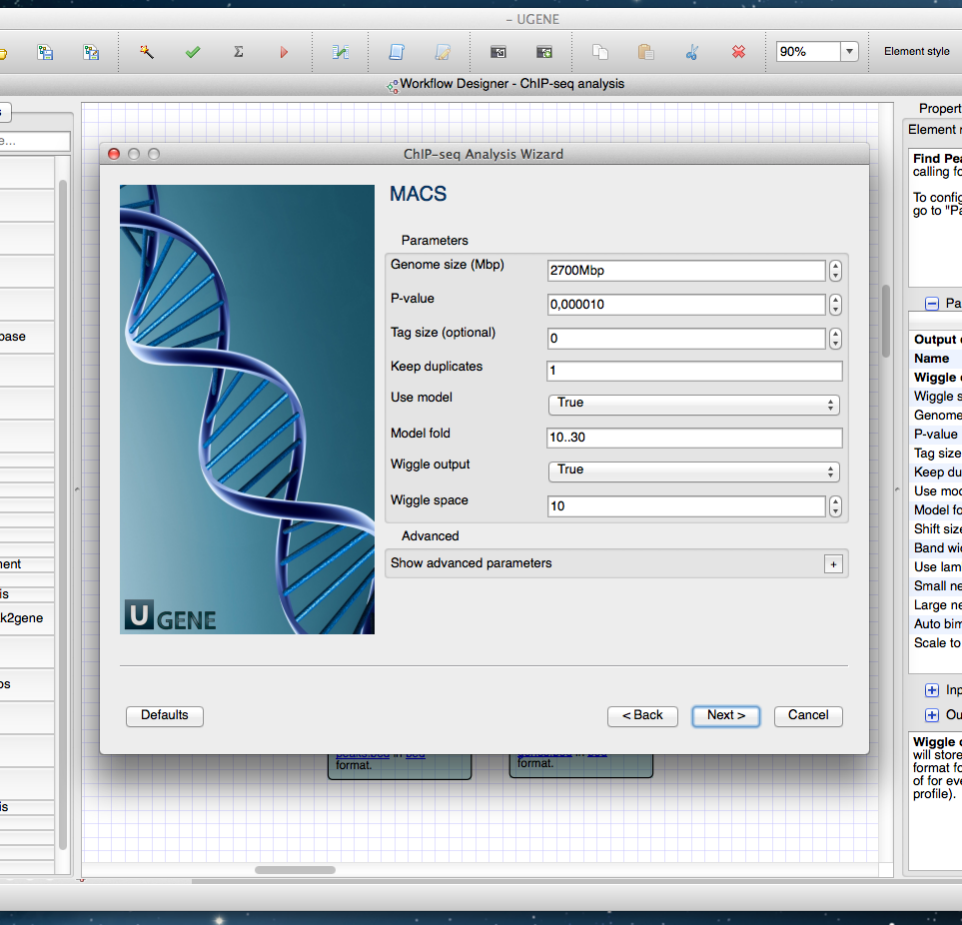
Wizard. A wizard page of the Cistrome pipeline is shown. On this page one can configure parameters of the MACS tool.

The wizards system is a helping layer for running NGS workflows. Simple forms (see for example Fig. 4) help users select the type of pre-built pipeline, setup parameters and inputs. It is possible to manage multiple input datasets (like RNA-seq paired-end tags) inside wizards. Thus, any NGS analysis pipeline can be run directly from the wizard. Advanced users are able to use the standard workflow interface with blocks and arrows, i.e., they can configure parameters directly in the Workflow Designer window, without the use of the wizards (see Figs. 1–3).

### Validation

The validation system checks if the designed pipeline can be run and generates warning or error messages. For instance, the system validates the presence of the required tools and data and immediately points out critical errors, if any.

### Dashboards

Each execution of a workflow creates its own context. The context stores the original workflow with filled parameters, statistics and output files, and so on.

**Figure 5:**
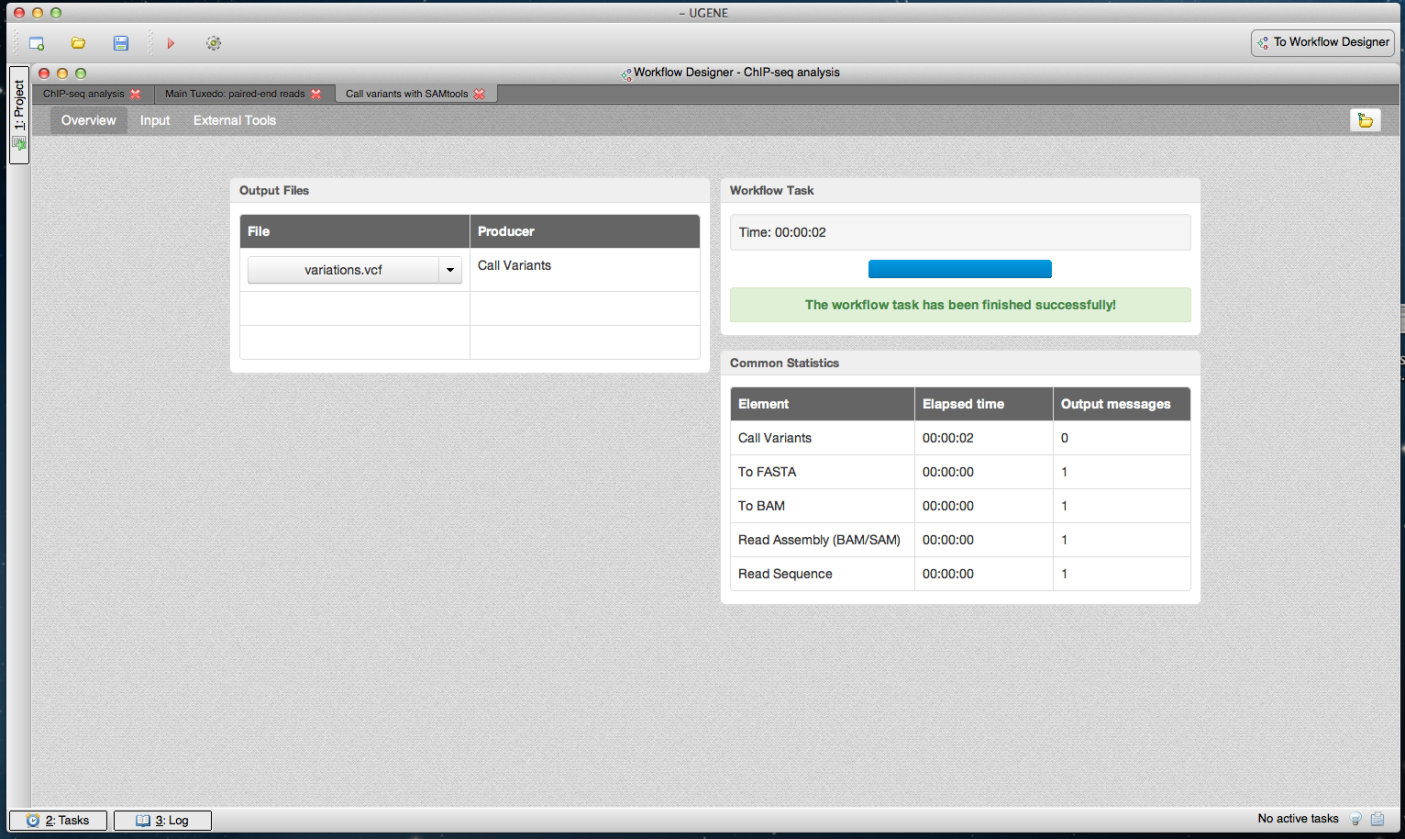
SAMtools workflow results in dashboard. A dashboard window with a result from running the SAMtools pipeline is shown. The “Overview” page of the dashboard is opened. It contains a link to the output variants file.

**Figure 6:**
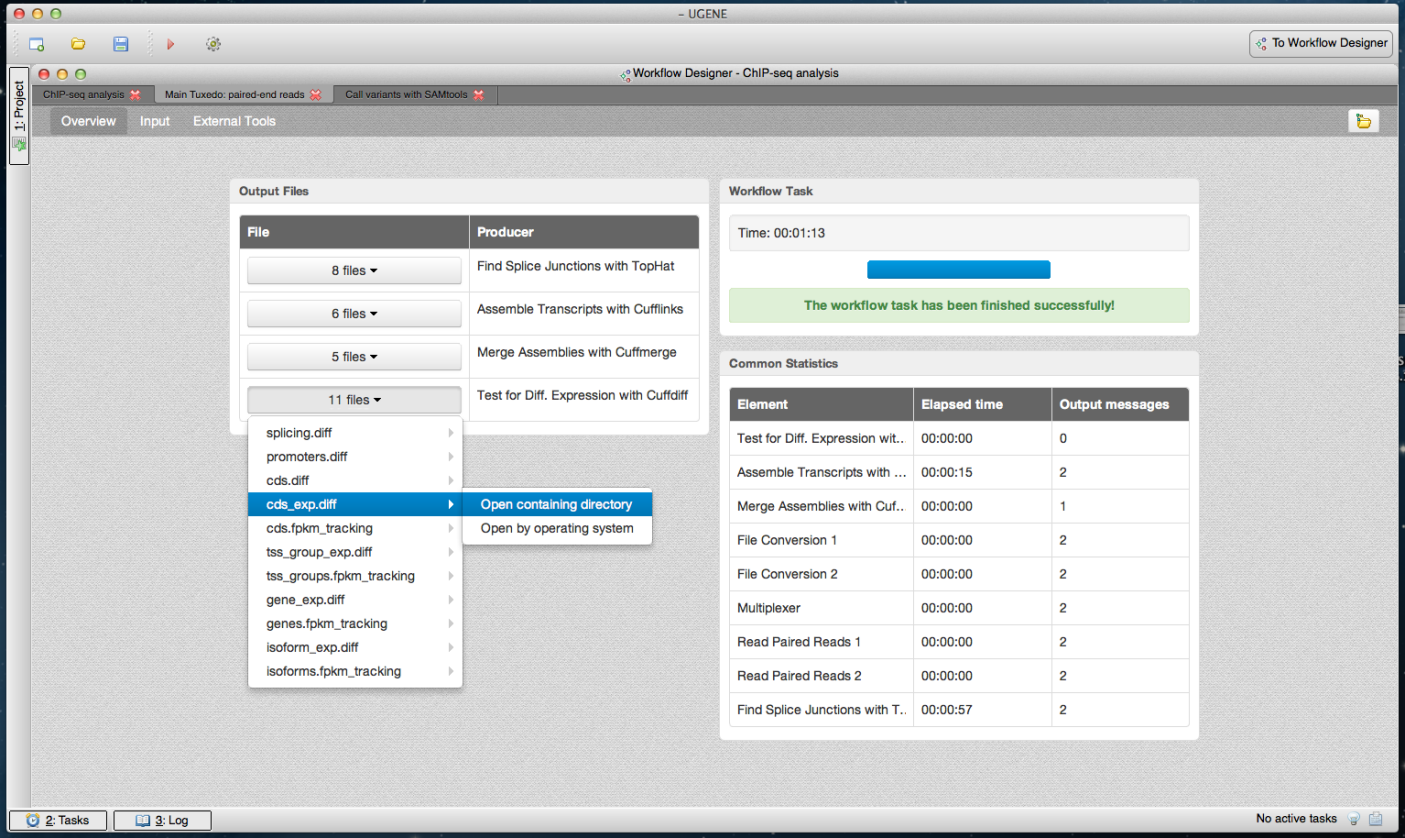
Tuxedo workflow results in dashboard. A dashboard window with the result of running the Tuxedo pipeline is shown. The “Overview” page of the dashboard is opened. The output files are grouped by the workflow elements that produced the output. One of the groups with 11 result files is opened. A user can open a result file in UGENE by clicking on it in the dashboard. Alternatively, each file can be opened outside UGENE (i.e., it can be opened by operating system) or the directory that contains the file can be opened directly from the dashboard.

**Figure 7:**
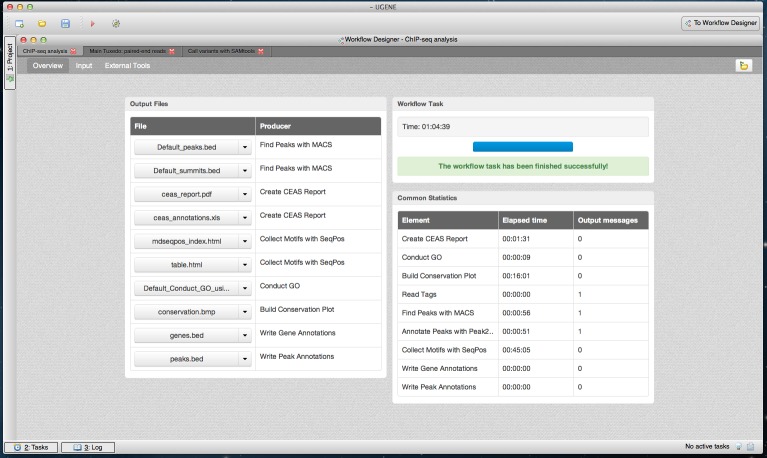
Cistrome workflow results in dashboard. A dashboard window with the result of running the Cistrome pipeline is shown. The “Overview” page of the dashboard is opened.

The dashboard appears after the workflow is started. As workflow results are calculated, the dashboard is filled with information such as the execution progress, links to the output files and common statistics per each workflow element, such as elapsed time (Figs. 5–7).

**Figure 8:**
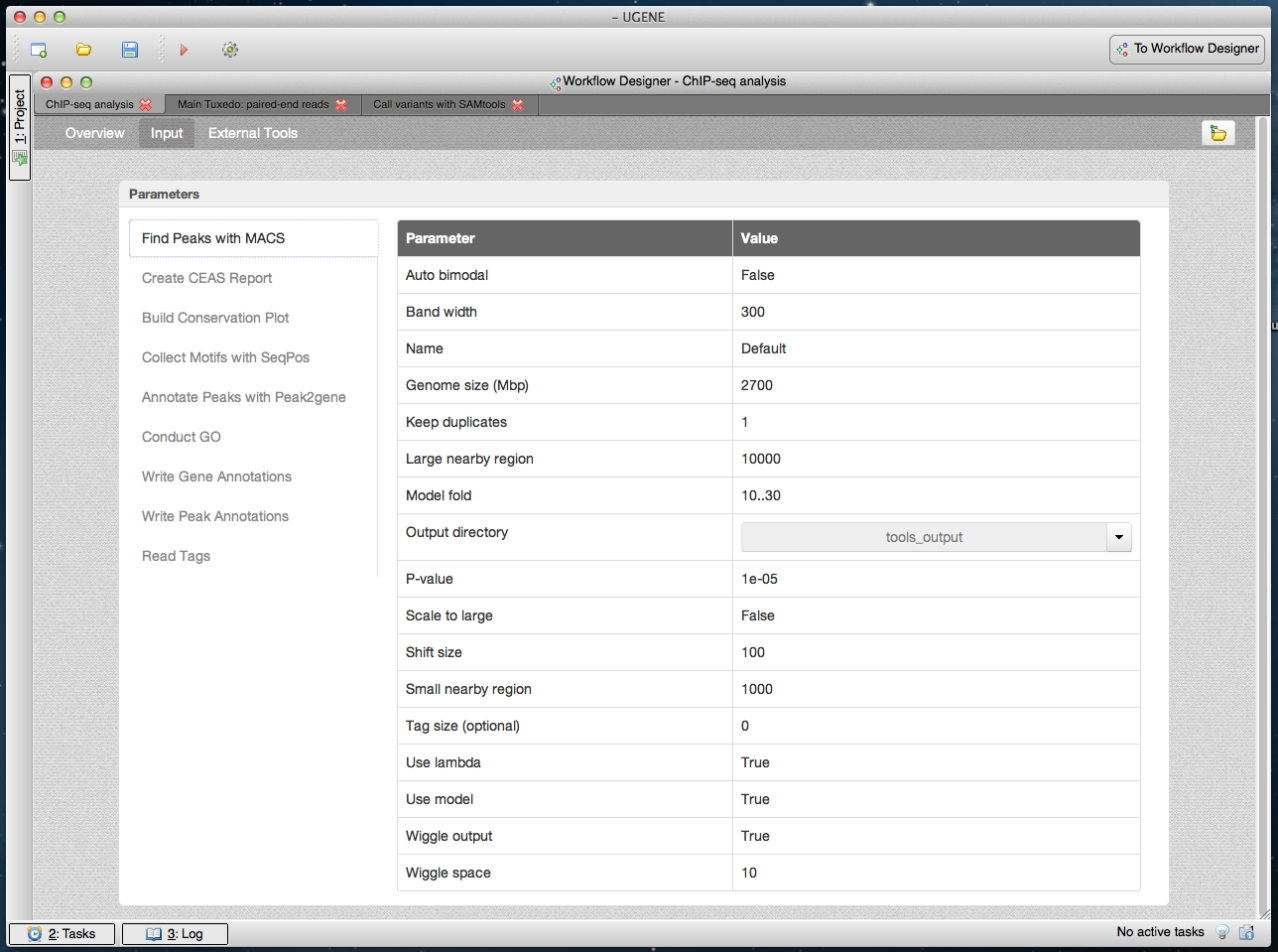
Cistrome result in dashboard: MACS input parameters. A dashboard window with the result of running the Cistrome pipeline is shown. The “Input” page of the dashboard is opened. Input parameters that were used to run the pipeline are shown. Parameters of the “Find Peaks with MACS” element are currently selected.

**Figure 9:**
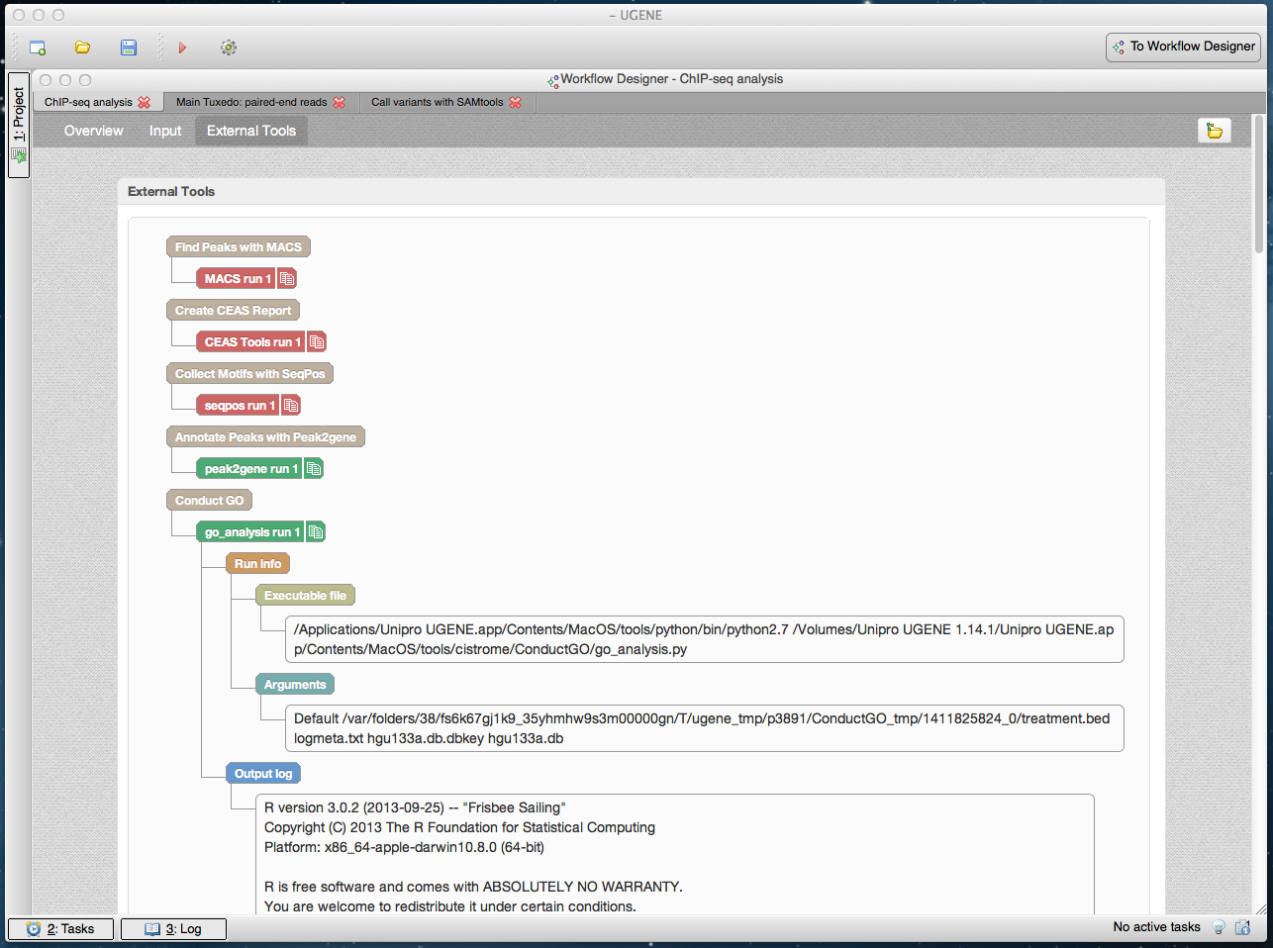
Cistrome result in dashboard: details about the tools used. A dashboard window with the result of running the Cistrome pipeline is shown. The “External Tools” page of the dashboard is opened. It contains details about the external tools runs (MACS, CEAS, seqpos, etc.). The details about the “go_analysis” tool are expanded.

The dashboard also contains information about the input parameters (Fig. 8). Advanced users can browse details about the tools’ execution: executable files, command-line arguments and logs (Fig. 9).

The dashboards are stored between launches of UGENE, so a user can browse results or load the original workflow at any time.

### System requirements and technical details

Recommended system requirements to run the pipelines are at least 2Gb of RAM and enough disk space to store the NGS data and the UGENE package (https://ugene.unipro.ru/wiki/display/UUOUM/System+Requirements). However, UGENE, when possible, makes use of additional RAM and multi-core capabilities of an environment. In particular, if a tool in a pipeline has a parameter to be run in multiple threads, then the value of the parameter is automatically set by UGENE to the number of cores of the computer.

In addition, the Cistrome pipeline optionally requires Internet connection. This is required for the ConductGo tool that sends a request to an external database DAVID (http://david.abcc.ncifcrf.gov/).

One should select a UGENE package to download depending on the operating system of the target computer and the required type of analysis.

• For running the variant calling and the Tuxedo pipelines a full UGENE package is enough. Note that a Unix-like system is currently required for the Tuxedo pipeline, as TopHat and some of the Cufflinks tools are available on the Unix-like systems only.
• For running the Cistrome pipeline one should download an NGS UGENE package, as it contains additional data internally used by the Cistrome tools.

All UGENE packages are available for download on the UGENE downloads page (http://ugene.unipro.ru/download.html).

### Pipelines execution time

The execution time of the integrated pipeline tools depends on the input NGS data. For example, on a computer with 2.9 GHz Intel processor with 4 cores and 16 GB RAM, the approximate execution times are the following:

• Variant calling pipeline with hg19 chromosome 11 from UCSC (http://genome.ucsc.edu/) as the reference sequence and chromosome 11 alignment with NA20887 identifier (Gujarati Indian from Houston, Texas) from the 1000 Genomes Project (http://www.1000genomes.org/): 15 min
• Tuxedo pipeline with RNA-seq data from human cell lines as specified in the Tuxedo protocol: 8.5 h
• Cistrome pipeline using ENCODE ChIP-seq experiment data (http://genome.ucsc.edu/ENCODE/dataMatrix/encodeChipMatrixHuman.html) with “H1-hESC” cell type and “REST” transcription factor, lab provided identifier: SL978: 6.5 h

The execution time is comparable to the time of the original tools themselves. A detailed calculation and the comparison can be found in the additional file.

## Conclusions

We incorporated into UGENE a set of popular NGS pipelines and tools so one can use both the ready pipeline samples or create custom ones. All tools and pipelines are available through the UGENE graphical user interface. Additional infrastructure was developed in UGENE to simplify configuration of the pipelines and manage the results of different pipeline runs.

For each supported operating system we created an NGS package that is easy to set up for a biologist. The packages contain all the needed tools and data out of the box and require no additional configuration.

## Supplemental Information

10.7717/peerj.644/supp-1 Supplemental Information 1 Execution time comparison Comparison of the execution time of the NGS tools integrated into pipelines in UGENE with the time of the original tools themselves.

## References

[ref-1] Goble CA, Bhagat J, Aleksejevs S, Cruickshank D, Michaelides D, Newman D, Borkum M, Bechhofer S, Roos M, Li P, De Roure D (2010). myExperiment: a repository and social network for the sharing of bioinformatics workflows. Nucleic Acids Research.

[ref-2] Goecks J, Nekrutenko A, Taylor J, The Galaxy Team (2010). Galaxy: a comprehensive approach for supporting accessible, reproducible, and transparent computational research in the life sciences. Genome Biology.

[ref-3] Langmead B, Salzberg S (2012). Fast gapped-read alignment with Bowtie 2. Nature Methods.

[ref-4] Langmead B, Trapnell C, Pop M, Salzberg SL (2009). Ultrafast and memory-efficient alignment of short DNA sequences to the human genome. Genome Biology.

[ref-5] Li H, Durbin R (2009). Fast and accurate short read alignment with Burrows-Wheeler transform. Bioinformatics.

[ref-6] Li H, Handsaker B, Wysoker A, Fennell T, Ruan J, Homer N, Marth G, Abecasis G, Durbin R, 1000 Genome Project Data Processing Subgroup (2009). The sequence alignment/map format and SAMtools. Bioinformatics.

[ref-7] Liu T, Ortiz JA, Taing L, Meyer CA, Lee B, Zhang Y, Shin H, Wong SS, Ma J, Lei Y, Pape UJ, Poidinger M, Chen Y, Yeung K, Brown M, Turpaz Y, Liu XS (2011). Cistrome: an integrative platform for transcriptional regulation studies. Genome Biology.

[ref-8] Liu XS, Brutlag DL, Liu JS (2002). An algorithm for finding protein-DNA binding sites with applications to chromatin-immunoprecipitation microarray experiments. Nature Biotechnology.

[ref-9] Matys V, Kel-Margoulis OV, Fricke E, Liebich I, Land S, Barre-Dirrie A, Reuter I, Chekmenev D, Krull M, Hornischer K, Voss N, Stegmaier P, Lewicki-Potapov B, Saxel H, Kel AE, Wingender E (2006). TRANSFAC and its module TRANSCompel: transcriptional gene regulation in eukaryotes. Nucleic Acids Research.

[ref-10] Okonechnikov K, Golosova O, Fursov M, UGENE team (2012). Unipro UGENE: a unified bioinformatics toolkit. Bioinformatics.

[ref-11] Portales-Casamar E, Thongjuea S, Kwon AT, Arenillas D, Zhao X, Valen E, Yusuf D, Lenhard B, Wasserman WW, Sandelin A (2009). JASPAR 2010: the greatly expanded open-access database of transcription factor binding profiles. Nucleic Acids Research.

[ref-12] Trapnell C, Pachter L, Salzberg SL (2009). TopHat: discovering splice junctions with RNA-Seq. Bioinformatics.

[ref-13] Trapnell C, Roberts A, Goff L, Pertea G, Kim D, Kelley DR, Pimentel H, Salzberg SL, Rinn JL, Pachter L (2012). Differential gene and transcript expression analysis of RNA-seq experiments with TopHat and Cufflinks. Nature Protocols.

[ref-14] Trapnell C, Williams BA, Pertea G, Mortazavi AM, Kwan G, van Baren MJ, Salzberg SL, Wold B, Pachter L (2010). Transcript assembly and quantification by RNA-Seq reveals unannotated transcripts and isoform switching during cell differentiation. Nature Biotechnology.

[ref-15] Wolstencroft K, Haines R, Fellows D, Williams A, Withers D, Owen S, Soiland-Reyes S, Dunlop I, Nenadic A, Fisher P, Bhagat J, Belhajjame K, Bacall F, Hardisty A, Nieva de la Hidalga A, Balcazar Vargas MP, Sufi S, Carole Goble C (2013). The Taverna workflow suite: designing and executing workflows of Web Services on the desktop, web or in the cloud. Nucleic Acids Research.

[ref-16] Xie Z, Hu S, Blackshaw S, Zhu H, Qian J (2009). hPDI: a database of experimental human protein-DNA interactions. Bioinformatics.

[ref-17] Zhang Y1, Liu T, Meyer CA, Eeckhoute J, Johnson DS, Bernstein BE, Nusbaum C, Myers RM, Brown M, Li W, Liu XS (2008). Model-based analysis of ChIP-Seq (MACS). Genome Biology.

[ref-18] Zhu C, Byers KJ, McCord RP, Shi Z, Berger MF, Newburger DE, Saulrieta K, Smith Z, Shah MV, Radhakrishnan M, Philippakis AA, Hu Y, De Masi F, Pacek M, Rolfs A, Murthy T, Labaer J, Bulyk ML (2009). High-resolution DNA-binding specificity analysis of yeast transcription factors. Genome Research.

