# Supplementary material for: Unipro UGENE NGS pipelines and components for variant calling, RNA-seq and ChIP-seq data analyses
Source: PeerJ. 2014 Nov 4;2:e644. doi: 10.7717/peerj.644 (PMC4226638; doi:10.7717/peerj.644)
Supplement: Supplemental Information 1 — Comparison of the execution time of the NGS tools integrated into pipelines in UGENE with the time of the original tools themselves. [file peerj-02-644-s001.pdf]

Execution time comparison

An additonal file for manuscript "Unipro UGENE NGS pipelines and components for variant calling, RNA-seq and ChIP-seq data analyses

The manuscript describes NGS pipelines integrated into the Unipro UGENE (Okonechnikov et al., 2012) desktop toolkit. The pipelines consist of popular open-source (command-line) tools.

This document contains comparison of the execution time of the integrated tools with the time of the original tools themselves.

All calculations were performed on the same machine with the following characteristics:

- Processor: 2.9 GHz Intel (with 4 cores)
- Memory: 16 GB 1600 MHz DDR3
- Operating system: Mac OS X 10.9.2

The results summary is specified in Table 1. Tables 2, 3, and 4 contains results for pipelines "Variant Calling with SAMtools", "Tuxedo Pipeline for RNA-seq Data Analysis", and "Cistrome Pipeline for ChIP-seq Data Analysis" correspondingly.

Tables

	**Execution time in UGENE**	**Execution time of the original tools**
**Variant calling pipeline**	15:09	14:13
**RNA-seq pipeline**	8:26:00	8:12:48
**ChIP-seq pipeline**	6:13:32	5:56:51

Table 1. Execution time summary for the three pipelines and the original tools

**Tools versions**	
**Tool**	**Version**
SAMtools package (the version is common for all utilities)	0.1.19
Perl scripts interpreter	5.12.4
UGENE NGS Package	1.13.2
**Commands and execution time**	
**Command**	**Time**
samtools mpileup –uf <*input_reference*> <*input_bam*> | bcftools view –vg – | vcfutils.pl varFilter –D 100	14:13
**Total execution time of the original tools:**	**14:13**
**Total execution time of the pipeline in UGENE:**	**15:09**

Table 2. Execution time of the "Variant Calling with SAMtools" pipeline in UGENE and the original tools. The calculations were performed using hg19 chromosome 11 from UCSC (http://genome.ucsc.edu/) as the reference sequence and chromosome 11 alignment with NA20887 identifier (Gujarati Indian from Houston, Texas) from the 1000 Genomes Project (http://www.1000genomes.org/) as the input BAM file. Besides the execution time results, the table specifies tools, their versions, and commands used to launch the original tools.

<table>
<tr><th colspan="3">Tools versions</th></tr>
<tr><th colspan="2">Tool</th><th>Version</th></tr>
<tr><td colspan="2">SAMtools package</td><td>0.1.19</td></tr>
<tr><td colspan="2">Bowtie2</td><td>2.1.0</td></tr>
<tr><td colspan="2">TopHat</td><td>2.0.9</td></tr>
<tr><td colspan="2">Cufflinks tools (gffread, cuffmerge, cufflinks, cuffdiff, cuffcompare)</td><td>2.0.2</td></tr>
<tr><td colspan="2">Python scripts interpreter</td><td>2.7.5</td></tr>
<tr><td colspan="2">UGENE NGS Package</td><td>1.13.2</td></tr>
<tr><th colspan="3">Commands and execution time</th></tr>
<tr><th>Tool</th><th>Command</th><th>Time</th></tr>
<tr><td rowspan="6">TopHat</td><td>tophat –p 4 –G genes.gtf –o C1_R1_thout genome C1_R1_1.fq C1_R1_2.fq</td><td>25:17</td></tr>
<tr><td>tophat –p 4 –G genes.gtf –o C1_R2_thout genome C1_R2_1.fq C1_R2_2.fq</td><td>25:17</td></tr>
<tr><td>tophat –p 4 –G genes.gtf –o C1_R3_thout genome C1_R3_1.fq C1_R3_2.fq</td><td>35:24</td></tr>
<tr><td>tophat –p 4 –G genes.gtf –o C2_R1_thout genome C2_R1_1.fq C2_R1_2.fq</td><td>25:21</td></tr>
<tr><td>tophat –p 4 –G genes.gtf –o C2_R2_thout genome C2_R2_1.fq C2_R2_2.fq</td><td>25:24</td></tr>
<tr><td>tophat –p 4 –G genes.gtf –o C2_R3_thout genome C2_R3_1.fq C2_R3_2.fq</td><td>25:23</td></tr>
<tr><td rowspan="6">Cufflinks</td><td>cufflinks –p 4 –o C1_R1_clout C1_R1_thout/accepted_hits.bam</td><td>11:21</td></tr>
<tr><td>cufflinks –p 4 –o C1_R2_clout C1_R2_thout/accepted_hits.bam</td><td>11:07</td></tr>
<tr><td>cufflinks –p 4 –o C1_R3_clout C1_R3_thout/accepted_hits.bam</td><td>11:27</td></tr>
<tr><td>cufflinks –p 4 –o C2_R1_clout C2_R1_thout/accepted_hits.bam</td><td>11:33</td></tr>
<tr><td>cufflinks –p 4 –o C2_R2_clout C2_R2_thout/accepted_hits.bam</td><td>11:12</td></tr>
<tr><td>cufflinks –p 4 –o C2_R3_clout C2_R3_thout/accepted_hits.bam</td><td>11:28</td></tr>
<tr><td>Cuffmerge</td><td>cuffmerge –g genes.gtf –s genome.fa –p 4 assemblies.txt</td><td>4:42</td></tr>
<tr><td>Cuffdiff</td><td>cuffdiff –o diff_out –b genome.fa –p 4 –L C1,C2 –u merged_asm/merged.gtf ./C1_R1_thout/accepted_hits.bam,./C1_R2_thout/accepted_hits.bam, ./C1_R3_thout/accepted_hits.bam ./C2_R1_thout/accepted_hits.bam,./C2_R2_thout/accepted_hits.bam, ./C2_R3_thout/accepted_hits.bam</td><td>4:17:52</td></tr>
<tr><th colspan="2">Total execution time of the original tools:</th><th>8:12:48</th></tr>
<tr><th colspan="2">Total execution time of the pipeline in UGENE:</th><th>8:26:00</th></tr>
</table>

Table 3. Execution time of the “Tuxedo Pipeline for RNA-seq Data Analysis” pipeline in UGENE and the original tools. The calculations were performed using data specified in (Trapnell et al., 2012): reads (in FASTQ format) at accession [GEO: GSE32038], genome and annotations from the fruit fly iGenome package. Besides the execution time results, the table specifies tools, their versions, and commands used to launch the original tools.

**Tools versions**	
**Tool**	**Version**
MACS	1.4.2
CEAS	0.9.9.7 (package version 1.0.2)
Conservation plot	0.1
MDSeqPos	2.0
Peak2Gene	1.02
Conduct GO	1.0
Python scripts interpreter	2.7.5
R scripts interpreter	3.0.2
UGENE NGS Package	1.13.2

**Commands and execution time**		
**Tool**	**Command**	**Time**
MACS	macs14.py –t <*input_treatment_file*> –wig —single-profile	9:30
CEAS	ceas.py –b <*macs_peaks_output*> –w <*macs_wig_output*> –g <*hg19_refSeq*>	16:22
Conservation plot	conservation_plot.py –d <*dir_to_store_phastcons_scores*> —height=1000 —width=1000 <*macs_summits_output*>	1:41:11
SeqPos	MDSeqPos.py <*macs_summits_output*> <*hg19_genome*> –m cistrome.xml –v	3:46:29
Peak2Gene	peak2gene.py –treat <*macs_summits_output*> —op=all –d 3000 –g <*hg19_refSeq*> –e <*geneIdTranslations*>	1:10
Conduct GO	go_analysis.py <*title*> <*peak2gene_output*> <*output_file*> <*hgu133a_gene_universe*>	2:09
**Total execution time of the original tools:**		**5:56:51**
**Total execution time of the pipeline in UGENE:**		**6:13:32**

Table 4. Execution time of the "Cistrome Pipeline for ChIP-seq Data Analysis" pipeline in UGENE and the original tools. The calculations were performed using ENCODE ChIP-seq experiment data (http://genome.ucsc.edu/ENCODE/dataMatrix/encodeChipMatrixHuman.html) with "H1-hESC" cell type and "REST" transcription factor, lab provided identifier: SL978. The data were converted to BED format using BEDtools. Besides the execution time results, the table specifies tools, their versions, and commands used to launch the original tools.

References

Okonechnikov K, Golosova O, Fursov M, UGENE team. 2012. Unipro UGENE: a unified bioinformatics toolkit. *Bioinformatics* 28:1166–1167.

Trapnell C, Roberts A, Goff L, Pertea G, Kim D, Kelley DR, Pimentel H, Salzberg SL, Rinn JL, Pachter L. 2012. Differential gene and transcript expression analysis of RNA-seq experiments with TopHat and Cufflinks. Nature Protocols 7:562–578.
